# Multitasking in Driving as Optimal Adaptation Under Uncertainty

**DOI:** 10.1177/0018720820927687

**Published:** 2020-07-30

**Authors:** Jussi P. P. Jokinen, Tuomo Kujala, Antti Oulasvirta

**Affiliations:** 1 174277 Aalto University, Finland; 2 4168 University of Jyväskylä, Finland; 3 Finnish Center for Artificial Intelligence FCAI, Finland

**Keywords:** driving, multitasking, task interleaving, computational rationality, reinforcement learning

## Abstract

**Objective:**

The objective was to better understand how people adapt multitasking behavior when circumstances in driving change and how safe versus unsafe behaviors emerge.

**Background:**

Multitasking strategies in driving adapt to changes in the task environment, but the cognitive mechanisms of this adaptation are not well known. Missing is a unifying account to explain the joint contribution of task constraints, goals, cognitive capabilities, and beliefs about the driving environment.

**Method:**

We model the driver’s decision to deploy visual attention as a stochastic sequential decision-making problem and propose hierarchical reinforcement learning as a computationally tractable solution to it. The supervisory level deploys attention based on per-task value estimates, which incorporate beliefs about risk. Model simulations are compared against human data collected in a driving simulator.

**Results:**

Human data show adaptation to the attentional demands of ongoing tasks, as measured in lane deviation and in-car gaze deployment. The predictions of our model fit the human data on these metrics.

**Conclusion:**

Multitasking strategies can be understood as optimal adaptation under uncertainty, wherein the driver adapts to cognitive constraints and the task environment’s uncertainties, aiming to maximize the expected long-term utility. Safe and unsafe behaviors emerge as the driver has to arbitrate between conflicting goals and manage uncertainty about them.

**Application:**

Simulations can inform studies of conditions that are likely to give rise to unsafe driving behavior.

## Introduction

Interactive technologies, such as smartphones and in-car information and entertainment systems, can serve a driver in various ways but may also influence the ability to operate the vehicle safely. While an interface may be easy to use in single-task conditions, it can be distractive when the user must timeshare attention with driving (Noy et al., 2004). Multitasking and task interleaving (switching between concurrently conducted tasks) behind the wheel is one of the most frequent contributors to driving accidents (Dingus et al., 2016). A key reason for this is that attending a secondary (nondriving) task quickly results in increased uncertainty about the states of the driving task, such as the current lane position (Horrey et al., 2006). One critical question for human factors research is how drivers adapt to the uncertainty associated with multitasking, given particular factors in the driving environment and elements of in-car interactions’ design.

This paper presents a computational model in which multitasking is formalized as optimal adaptation under uncertainty. We consider the driver as a computationally rational agent operating in a task environment that constrains the said agent’s behavior, resulting in bounded optimal adaptation (Gershman et al., 2015; Howes et al., 2009; Simon, 1969). In our model, multitasking behavior emerges as adaptation to cognitive and task bounds, permitting the investigation of multitasking in various combinations of driving and in-car tasks.

In multitasking, two or more interleaved tasks compete for limited information-processing resources, such as visual attention (Meyer & Kieras, 1997; Salvucci & Taatgen, 2008). Any tasks unattended by the agent have limited or no observability, and decreased task performance often follows. The multitasking agent encounters an optimization problem: how to allocate limited resources to maximize joint task performance (Navon & Gopher, 1979). The solution must consider the tasks’ attentional demands and what could be expected to be achieved if attention is shifted. Hence, our goal is a computational model of multitasking in driving that explains and predicts multitasking behavior as optimal adaptation, thereby offering a tool for researchers and practitioners. Models of this sort incorporate explicit theoretical assumptions and permit testing against empirical results. Generating adaptive behavior, they can be used to help design new technologies that encourage safer driving. They can also be used to explore complex interactions between the parameters of the driving task and those of the in-car interface without the need to resort to costly experiments.

Driver behavior is known to change in response to traffic, driving environment, in-car user interface design, idiosyncratic traits, and other elements. Interleaving decisions can be assumed to result from adaptation under uncertainty: the human visual system is able to sample accurately from only a small subset of the visual field, and the task environment contains dynamics that can be difficult or impossible to predict. Hence, drivers do not obey fixed rules for switching tasks, but consider the probable consequences of switching. For instance, Wierwille’s (1993) visual sampling model of driving suggests that, on average, drivers adjust their off-road glance durations between 0.5 and 1.5 s as the dynamic demands of traffic dictate. Drivers tend to prolong in-car glances to reach natural breakpoints, such as subtask boundaries, for instance to complete visual search (Janssen et al., 2012; Lee et al., 2012). Furthermore, drivers’ multitasking has displayed strategic changes in task performance, based on variations in the workload and the priority of particular task-specific goals (Cnossen et al., 2000). Also, the skill of the individual clearly affects multitasking strategies (Janssen & Brumby, 2015). However, we are lacking a unified account of the mechanisms behind these adaptive behaviors (Gutzwiller et al., 2019).

### Computational Models of Multitasking

The EV and SEEV (salience, effort, expectancy, value) models predict experts’ visual sampling from task-relevant information sources (Sheridan, 1970; Wickens et al., 2003). These models are based on the idea of expected information bandwidth as one of the key factors determining the optimal visual sampling frequency for a channel (Senders, 1964), together with the value functions of the related tasks and a threshold of acceptable uncertainty about the state of the controlled system (Sheridan, 1970). For instance, in a lane-keeping task, the decision about making a corrective input with the steering wheel depends on information about the lane position, which contains uncertainty that depends on the driving conditions. A high-bandwidth condition, such as strong wind turbulence, increases uncertainty and thus requires more frequent visual sampling. The model is able to predict a driver’s safety-relevant behaviors based on the information requirements of the driving and the in-car task and the relative priority or value assigned to them by the driver. The approach has been utilized for predicting the effects of task value, uncertainty, and expectations on allocation of the focus of visual attention to in-car tasks (Horrey et al., 2006). One of the drawbacks of these models is that the overall determination of the expectancy and value associated with various tasks are based on heuristics from human experts.

Another class of computational visual sampling models emphasizes the uncertainty of task-relevant states (e.g., Sprague & Ballard, 2004; Sullivan et al., 2012; Tong et al., 2017). In these, similar to EV/SEEV models, uncertainty is related to the concept of the bandwidth of a continuous signal or expected error (Senders et al., 1967; Senders, 1964; Shannon, 1948). The model predicts that drivers shift attention based on the accumulating uncertainty about the driving task. A more demanding task may lead to postponing the secondary task (Jamson & Merat, 2005), but an incorrect estimation of this demand may also lead to unsafe driving. For instance, Lee and Lee (2019) have recently modeled drivers’ task interleaving as an outcome of uncertainty about the roadway and subtask boundaries. Close to these natural breakpoints, drivers may fail to suspend the task at the cost of safety. Typically, models in this class are unable to explain how the uncertainty of one’s task-relevant beliefs evolves and how behavior adapts to the dynamics of the task environment and varying subtask goals.

In cognitive architectures such as EPIC (Meyer & Kieras, 1997) and ACT-R (Anderson et al., 2004), the use of limited computational resources is directed by rules that specify how tasks are conducted. Models using these architectures are able to generate detailed simulations of task behavior, such as glances between interleaved tasks. The *theory of threaded cognition*, based on ACT-R, has been developed to understand and predict task interleaving in multitasking via the principle of autonomous task threads that share attentional resources (Salvucci & Taatgen, 2008). Each task thread has its own goal, reserving and freeing attentional resources in accordance with availability, demand, and urgency. This provides psychologically plausible constraints, and the model yields predictions with good fit to aggregate human data (Salvucci & Taatgen, 2008), including drivers’ multitasking performance (Kujala & Salvucci, 2015). The model can be used to predict how the design parameters of the in-car interface impact multitasking strategies and driving safety. While this approach formalizes the impact of task constraints on multitasking behavior, it does not explain the emergence of adaptive strategies, which often must be formulated as simple and fixed rules. As a result, the approach does not predict how task-interleaving behavior adapts to changes in uncertainty related to the task states.

Cognitive constraint modeling (CCM) complements cognitive architecture–based models of multitasking (Howes et al., 2004; Janssen et al., 2012). Instead of focusing on the cognitive architecture in an attempt to reproduce the internal psychological mechanisms of multitasking, the goal is to predict performance from the constraints of the task environment (Brumby et al., 2007). Possible interleaving strategies are determined by performance trade-off functions, dictated by a model of the environment, and optimal strategies in terms of task priority instructions. For instance, Brumby et al. (2007) have shown that people are able to adjust their task interleaving rationally by considering task constraints and prioritization. The CCM approach informs understanding of the variability of multitasking drivers’ strategies. This is accomplished by specifying how cognitive and task-related constraints affect behavior and computing how different multitasking strategy choices result in different task outcomes. Such modeling is silent, however, about how the strategy choices are made and how drivers dynamically adjust their behavior to particular task constraints.

Neither the threaded cognition nor the CCM approach formalizes decision making in driving under uncertainty caused by limited attention, which is a critical factor when several tasks compete for the same attentional resources. The literature reviewed has established that what constitutes optimal multitasking policy—a mapping from task states to action probabilities—should be defined in relation to the agent’s uncertainty about the goal-relevant task states. For instance, the driving conditions, such as speed, traffic, and visibility, all impact how much visual sampling is needed for the driver to have a reliable estimate of the car’s position on the road (possibly relative to traffic). Furthermore, the goals for the tasks should take the form of a value or utility function allowing the agent to weigh the relevance of the concurrent tasks. What prior literature does not sufficiently address is how a multitasking agent adapts the task-interleaving policy to the variable cognitive and environmental constraints of the tasks in an attempt to maximize the expected joint task gain.

### Goals of This Paper

Viewing human behavior as optimal adaptation to constraints in conditions of uncertainty, we developed a model of multitasking in driving. The model presented in this paper addresses the problem of optimal attention allocation in terms of a policy, which dictates which actions are suitable in various situations. An optimal policy maximizes expected (joint) task gain. We assume that this optimal policy emerges from the task and cognitive bounds, including the uncertainty resulting from partial observability of the states and dynamics of the environment. This is achieved adaptively using a learning algorithm that attempts to optimize behavior by preferring chains of actions that generally result in desired outcomes. In our task environment, these outcomes are safe driving and efficient performance in the interleaved task. Given the specifics of these tasks as well as how accurate beliefs the agent is able to form about the states of the task environment, the discovered behavior can be said to approximate boundedly optimal behavior.

Our model architecture utilizes a hierarchical structure, decomposing the multitasking scenario into a series of task environments, goals, and corresponding policies. There is a driving task, wherein the goal is to keep the car within lane boundaries; a visual search task, with the goal of visually locating a target element among distractors; and a supervisory task, which allocates the limited visual attention resource between the two subtask models. The model has multiple bounds that compel its adaptive behavior. The driving task is bounded by how quickly and accurately the agent can update its belief as to the lane position and by how quickly and often the agent can act to adjust this position. Bounding the visual search task are the limits of the human visual system, which allow the visual encoding of only one element at a time and require time to move the eyes and process visual information. When not visually attending to the road, the model cannot observe the lane position (but can still steer), increasing uncertainty. Conversely, when the attention is on the road, the visual search task cannot progress, as uncertainty about the location of the visual target is not reduced. Finally, there is task-switching time in shifting attention between the road and the in-car display. Our model simulates adaptation to these bounds by learning a policy that manages uncertainty in the subtasks by allocating visual attention to the task that requires it the most.

Unlike those in some previous work, our model makes no assumptions about how long drivers should tend to the visual search task or when they should return to the driving one (e.g., Kujala & Salvucci, 2015). Instead, the glancing behavior is conceptualized as emerging when the agent’s optimal task-interleaving policy is resolved via reinforcement learning (RL; cf. Brumby et al., 2007). This behavior is sensitive to (1) the dynamically changing attentional demands of tasks (e.g., faster driving implying greater attentional demands), (2) the tasks’ relative value (e.g., the importance of driving safely vs. finding targets quickly), and (3) the constraints inherent to the agent (e.g., the duration of task switching, in which attention is on neither task). This sensitivity permits the model to predict the impact of environmental dynamics and cognitive architecture on the driver’s uncertainty and the resulting changes in driving behavior. Below we first present our model architecture and its computational principles. We then demonstrate how we validated the model’s predictions via a driving task with human participants. The model successfully predicts the effects of driving speed and search difficulty on driving performance, as represented by task time, lane deviation, and glancing behavior.

## Modeling

### A Hierarchical Model of Rational Multitasking

A *computationally rational* agent follows a policy that maximizes the expected gain or *utility* of its behavior, given the bounds of the task and the agent itself (Gershman et al., 2015; Howes et al., 2009). The first step in applications of computational rationality is to model the agent and task environment in a way that can account for these bounds and for the agent’s actions and goals (Howes, 2018). A *partially observable Markov decision process* (POMDP) serves as a flexible formalism that permits us to define an environment, consisting of states and a transition function; an agent, making partial observations of the environment and acting to change the state of the environment; and a reward function, reflecting the preferences of the agent (Kaelbling et al., 1998). Learning algorithms, such as those under the umbrella of *reinforcement learning*, are used to learn from interaction with the environment in order to find the optimal action policy, satisfying the condition of the agent behaving rationally, carrying out actions with the greatest expected utility (Sutton & Barto, 1998).

In the POMDP formalism, the agent performs sequential actions, maximizing the long-term gain through actions *a* ∈ A that create changes in the environment’s state *s* ∈ S (Kaelbling et al., 1998). These changes are governed by a transition function that specifies the probability T(s,a,s′)=p(s′∣s,a) of a resulting state *s′* given current state *s* and action *a*. Driving actions, for instance, include moving the steering wheel, which results in changes in lane position. The formalism also accounts for the agent’s incomplete information about aspects of the state of the world, by specifying an observation function that defines the probability O(s′,a,z′)=p(z′∣s′,a) of making an observation *z*′. As for the agent’s goals, the formalism includes a reward function *R*(*s*′), providing a real-valued reward for reaching a state *s*′. For instance, succeeding in an in-car search task yields a positive reward, while lane deviations carry a negative one. The agent modeled by a POMDP learns to perform actions that maximize the long-term cumulative reward. A policy π determines the choice of a new action, given a history of observations that are summarized as a belief *b*. An optimal policy π* maps beliefs to actions such that an agent following that policy maximizes the long-term cumulative reward. To model learning from experience, RL is employed to record how actions produce state changes and rewards; this process lets an experienced agent approximate an optimal policy (Sutton & Barto, 1998).

Our model is based on hierarchical organization of human cognitive control, which both offers neural plausibility and provides a computationally tractable solution for complex RL models (Botvinick, 2012; Frank & Badre, 2012). Hierarchical RL (HRL) can be used to break complex behavior into subtasks, which permits that the subtask policies are learned independently before subjugated to the supervisory control hierarchy. Each subtask has its own associated decision process, including the reward function. For instance, driving and in-car visual search subtasks have different goals and therefore reward functions. The key insight of this hierarchy is that the outcome of the multitasking episode is the joint function of the subtask reward functions. For instance, if the goal of the agent is to conduct visual search in an in-car interface while driving safely, the problem of the multitasking agent is to discover a task switch policy that minimizes lane excursions while maximizing search performance. The total reward of the hierarchical model is determined as the joint performance from these subtask models. The subtask reward functions can be changed to obtain policies for different goals, but also the joint multitasking reward function can be modified. For instance, relative weighing of how the subtask rewards are summed might result in the agent improving its visual search performance at the cost of safe driving.

**Figure 1:**
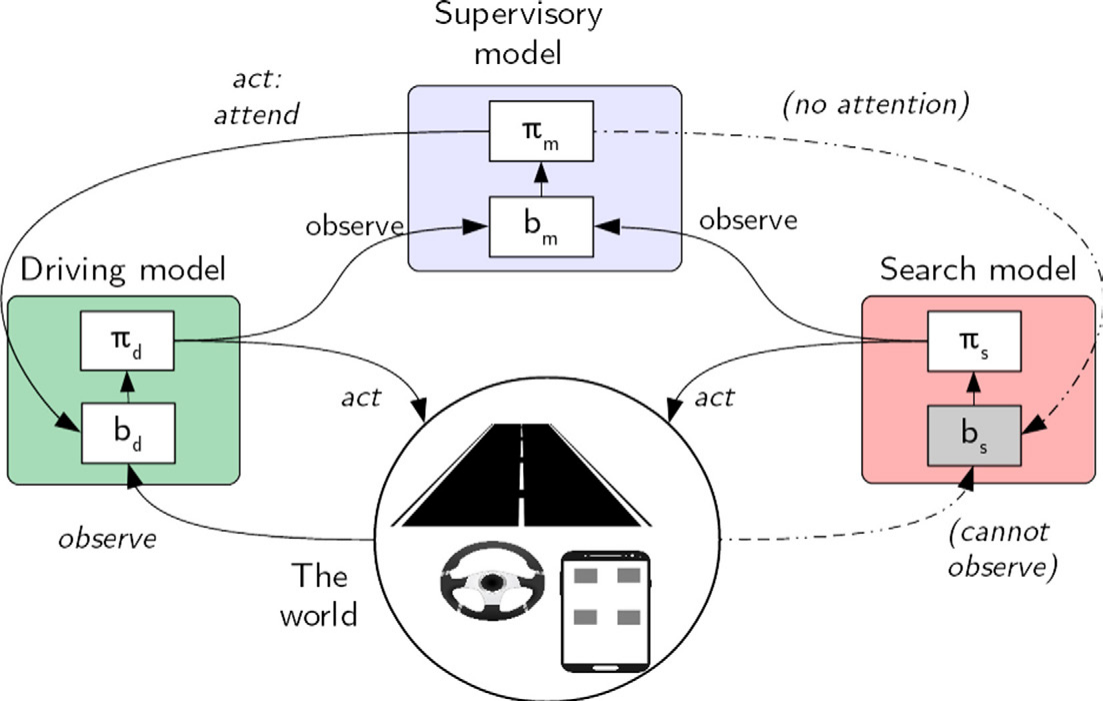
Our hierarchical multitasking model is composed of a driving model, a visual search model, and a supervisory model. The models’ actions are based on beliefs (*b*) about the world, given the policy learned (π). The subtask beliefs are updated via observations of the state of the world. The supervisory model assigns visual attention between tasks, granting the ability to make visual observations about the world. The supervisory model monitors the current subtask utility values, to track their attentional demands.

Our hierarchical model of multitasking is illustrated in Figure 1. We assume that there are independent task-specific models (e.g., for driving and in-car visual search) and a supervisory model that learns the optimal policy for shifting limited visual attention resources between these subtask models. Once RL has been applied for independently implementing and training the task-specific models, the supervisory model is trained to allocate attention between the trained task models. An important feature of our approach is that optimality must be understood in terms of bounded optimality, where the bounds and dynamics of the task environment and those of the cognitive architecture of the agent guide behavioral adaptation. Below, we briefly describe the task models. Full implementation details, addressing all parameter values, are explicated in the technical Appendix A, Algorithms 1–3. The model code is available at https://gitlab.com/jokinenj/multitasking-driving.

### Modeling Driving

For the driving model, we use a simple simulation *T_d_*(*s,a,s*′), wherein the car’s speed, the steering wheel’s position, and previous lane position *s* ∈ *S_d_* determine the next lane position, *s*′ ∈ *S_d_*. The agent can perform an action *a* ∈ *A_d_* to move the steering wheel so as to adjust the car’s position such that it remains within the specified limits. A negative reward *R_d_* is received when the car is beyond the specified lane bounds; the reward value is 0 within them. The agent utilizes a transition model, *T_d_*(*s,a,s*′), which approximates the true transition function *T_d_* and allows it to drive without visual attention for a short while. The agent’s belief *b_d_* is a distribution over possible lane positions *S_d_*. When the agent is able to make observations, *b_d_* is a joint posterior of *T_d_* and the observation function *O_d_*(*s*′,*a*,*o*). The belief that the car is in position *s*′ for a given action *a* ∈ *A_d_* is


(1) $$b\prime \left(s\prime \right)=\frac{O_{d}\left(s\prime ,a,o\right)\sum s\in S_{d}{}^{\tau_{d}\left(s,a,s\prime \right)b\left(s\right)}}{P_{r}\left(o|a,b\right)}$$

where *O_d_* is the probability of a correct observation. When attention is on the visual search task, the driving model is, in essence, “driving blind,” relying entirely on the transition model (*τ_d_*) and prior belief *b*.

The driving model is trained in two steps. First, it learns the transition model, *τ_d_*, by performing random actions and fully observing the outcomes. Hence, *τ_d_* becomes a stochastic prediction model of the physics of the driving task. After this, the model starts to drive, making observations and updating its belief about the car’s lane position by using Equation 1. For faster learning, we augment this belief distribution via a sufficient statistic, which in this case is the most likely road position and belief entropy.


(2,3) $$\bar{b}=\{{}_{-\overset{s}{\underset{s\in S}{\sum }}\left(b\left(s\right)\times \log\left(b\left(s\right)\right)\right)}^{arg\;max\;b\left(s\right)}$$

We use RL to train the model to an optimal policy $\pi_{d}^{*}$ , which for any *b̄* returns an action *a* ∈ *A_d_* that is expected to maximize the long-term reward. Specifically, we use SARSA (see Appendix A, Algorithm 1) to learn state–action utilities from previous state *s* ∈ *S* and action *a* ∈ *A*, the resulting reward *r*, and current state *s*′ ∈ *S* and current action *a*′ ∈ *A* (Sutton & Barto, 1998).


(4) Q(s,a)=Q(s,a)+α[r+γQ(s′,a′)−Q(s,a)],

where *α* is a learning constant and *γ* discounts future rewards. These *Q*-values indicate the utility or long-term expected gain of a particular action, for a given state. To balance between exploration and exploitation, we employ a *softmax action selection policy*, which selects actions on the basis of their relative *Q*-values. The probability of an action’s selection is


(5) $$p\left(a\right)=\frac{e^{Q\left(s,a\right)∕\tau }}{\sum_{i=1}^{n}e^{Q\left(s,i\right)∕\tau }},$$

where τ is a real-valued noise temperature greater than zero, controlling the exploration/exploitation trade-off.

### Modeling Visual Search

The visual search model simulates the eyes moving over an in-car graphical display. It is a POMDP wherein the state, *s* ∈ *S_s_*, is a discrete two-dimensional grid of visual elements. The model can take action *a* ∈ *A_s_* to fixate on any of these elements and encode it. The model’s belief is an initially empty grid that gets filled with the encoded visual elements. In addition, the belief encompasses the eyes’ fixation location. We use the EMMA model (Salvucci, 2001) to simulate eye movements. Time needed to encode a visual object is given as


(6) $$T_{e}=K\left[-log\left(f\right)\right]e^{k\cdot \in },$$

where *K* and *k* are encoding constants; *f* is the frequency of the object; and _∈_ is eccentricity, measured as the target’s distance from the current fixation. To reduce eccentricity and encoding time, the visual system may initiate a saccade. Saccade duration is


(7) $$T_{s}=t_{\text{prep}}+t_{\text{exec}}+Dt_{\text{sacc}},$$

where *t*_prep_, *t*_exec_, and *t*_sacc_ are constants related to the human visual system and *D* is the distance to be covered by the saccade, in degrees. If $T_{e}<t_{prep},$ the target is encoded without the eyes moving from the current fixation location. Otherwise, a saccade brings the fixation point close to the target, with the remaining encoding being conducted after this.

For each encoding of the target, the model receives a negative reward *R_s_* with a value equal to the encoding time. In addition, when the target is eventually found, the model gets a large positive reward. As with the driving model, we use RL to find an optimal policy $\pi_{s}^{*}$ , which for any combination of the current fixation location and the search-observation grid supplies a new target to be encoded. We use the same learning and action-selection equations as for the driving model.

### The Supervisory Model

The supervisory (multitasking) model is a POMDP wherein the state space contains the maximum *Q*-values for the current state of the two task models. This model applies two actions, *drive* and *search*, determining which subtask model receives attention. It learns an optimal attention-allocation policy $\pi_{m}^{*}$ , which maximizes the joint subtask reward *R_d_* + *R_s_*. The model is designed to maximize the number of search tasks completed in a given time while attempting to keep the cumulative reward for the driving task close to 0, which denotes making as few lane deviations as possible.

The multitasking model observes max *q_d_* and max *q_s_*: the current expected utility of the best available action for each of the two models. Together, these values and the task currently holding the attention form the current belief for the multitasking model. Based on the learned utilities, the model chooses an action, either to attend to driving or to search. If the previous action (referring to the task currently in focus) differs from the new one, a task-switch cost is added in the model (Allport et al., 1994). After this, if the selected action was to attend to driving, the driving model is run for one cycle of a small constant time while allowed to observe its lane position (Salvucci, 2006). If the action selected was to search, the search model is iterated once and driving is simulated forward without attention, for a duration matching the eye-movement action.

## Evaluation of the Model

### Method

Our model can be used to predict key performance variables in an in-car visual search task during driving. As all model parameters are either adapted from literature (e.g., eye movement and encoding times) or fixed to the task (e.g., car speed or the size of the in-car device), the goal of the evaluation was not to calibrate the model, but to see if the model predicts correctly the impact of different task conditions on the key performance variables. Correspondence of the predictions to human data is therefore the result of the realistic model specification, not tuning of parameters to the target data. Based on similar existing work (e.g., Horrey et al., 2006; Kujala & Salvucci, 2015; Wortelen et al., 2013), we designed an experiment to evaluate the model. Human participants used a driving simulator while searching for visual targets presented by an in-car display. The experiment employed a within-subjects 2×2 design with speed (60 and 120 km/hr) and number of search items per screen (6 and 9) as the independent variables. Condition order was counter-balanced over the entire sample. All data and analysis code are available at https://gitlab.com/jokinenj/multitasking-driving.

#### Participants

The participants were 12 volunteers (7 F, 5 M) selected via convenience sampling from responses to advertisements emailed to university mailing lists. Participants’ lifetime driving experience ranged from 10 to 29 years (*M* = 18, *SD* = 6) and age from 28 to 47 years (*M* = 36, *SD* = 5). All participants had normal or corrected-to-normal vision, and all admitted to using smartphones at least “infrequently” while driving. In monitoring throughout the experiment, none displayed symptoms of simulator sickness. The participants were compensated for their time with a cinema ticket. This research complied with the tenets of the Declaration of Helsinki. Informed consent was obtained from each participant.

#### Materials and procedure

**Figure 2:**
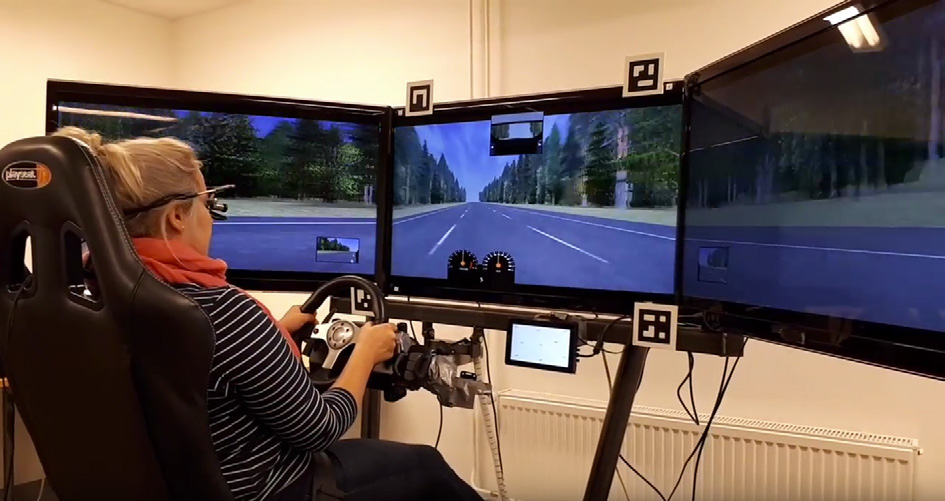
The experiment used a medium-fidelity driving simulator, with the in-car search task presented by a smaller display below the driving scene.

Our medium-fidelity driving simulator was composed of a Logitech G25 steering wheel and pedals, an adjustable driver’s seat, and three 40″ Samsung LED displays (4320 × 900) presenting the driving environment (Eepsoft driving simulation software, https://www.eepsoft.fi/; see Figure 2). A slightly leftward-bending highway road with three 3.5-m-wide lanes was selected for the study. The speed of the vehicle was fixed to make the task simpler for the humans to perform and to model computationally. To display the visual search task, a 7″ screen (1920 × 1080) was positioned below the main display, sufficiently close to minimize eye-movement time between the road vanishing point and the display but far enough away to inhibit observing the road during visual search (separation 34° of the visual field). The in-car visual search task involved searching for a target item among six or nine items. The task was designed to be prototypical of an everyday in-car search task, where the driver is searching for visual information on a display, such as a navigator, tablet, or a smartphone. For the purposes of controlling learning effects during the tasks, we stripped the task from semantic information, such as icons, and visual features, such as colors. The search items, three-letter combinations starting with “A” (e.g., “AKJ”), were displayed in black on a white background, in 28 pt Calibri type. The items were randomly distributed on the screen to rule out undesirable search strategies that were not incorporated into the search model. The search task was present on the screen for the whole duration of the trial. They were separated by at least 2° of visual angle, so that the participant had to encode each item with a separate fixation. There were 24 tasks (i.e., trials) in each task condition, with 12 foil (no-target) and 12 target-present screens (order randomized). One set of 24 tasks took approximately 90–150 s.

Gaze data were recorded with the Ergoneers Dikablis Essential eye-tracking system (sampling rate 50 Hz) and synchronized with the simulator log data (sampling rate 10 Hz). We used markers by Ergoneers to obtain data on where the eyes are in the task, while permitting the participants free movement of head (Figure 2). D-Lab software was used to automatically extract glance data from the recording.

The participants were allowed to practice the driving and in-car visual search tasks separately in the beginning of the experiment and, after eye-tracker calibration, they practiced a combination of the two. They were instructed to keep the car (i.e., the visible gauges; see Figure 2) in the center lane as accurately as possible while simultaneously searching the in-car screen visually. The search target was stated aloud by the experimenter before each task and remained the same throughout each trial (“AJK” or “AFN”). When the search item was found (target-present trial) or all items had been encoded (no-target trial), the participants reported finishing the trial by pulling a lever behind the steering wheel. This supplied the simulator log file with a timestamp. After this, they verbally reported whether the target was present to make sure that they were not cheating. The experimenter switched to the next task immediately after. This task instruction is similar to the specification of the reward function for the model, where any lane deviations are punished, and the model is rewarded for finding the search target as quickly as possible.

### Model Training and Validation

All adjustable model parameters were set on the basis of the literature or the human task conditions, apart from the action-related driving noise parameter *σ_d_* (Appendix A, Algorithm 1, L22), which was hand-tuned to approximate reliable but nondeterministic driving. No parameters were fitted to the observed human data; all but the task parameters were fixed across conditions, and all were justified by theory or the task. First, we trained the driving and search models separately for each of the speed and search-area conditions. Then, we trained the four supervisory models to correspond to each of the 2 × 2 conditions. We estimate the model’s fit for the following behavior metrics: trial time, *SD* of lateral offset from lane center, number of lane deviations, and number and duration of in-car glances. All metrics were aggregated within participants and then across participants, between conditions and task type (target present vs. “foil,” i.e., no target). We used various fit metrics to assess the model’s predictions, calculating each for all behavior metrics. First, we calculated the error in the model’s predictions relative to the human data, expressing this as absolute error, percentage error, and *SD* values from human data. Second, we regressed the human-data observations over the model’s predictions, reporting the linear model’s *R*^2^ for each behavior metric. These fit indices permit investigating how closely the model’s predictions fit the human data and how well the model reproduces the impact of the various conditions on performance.

### Results

**Figure 3:**
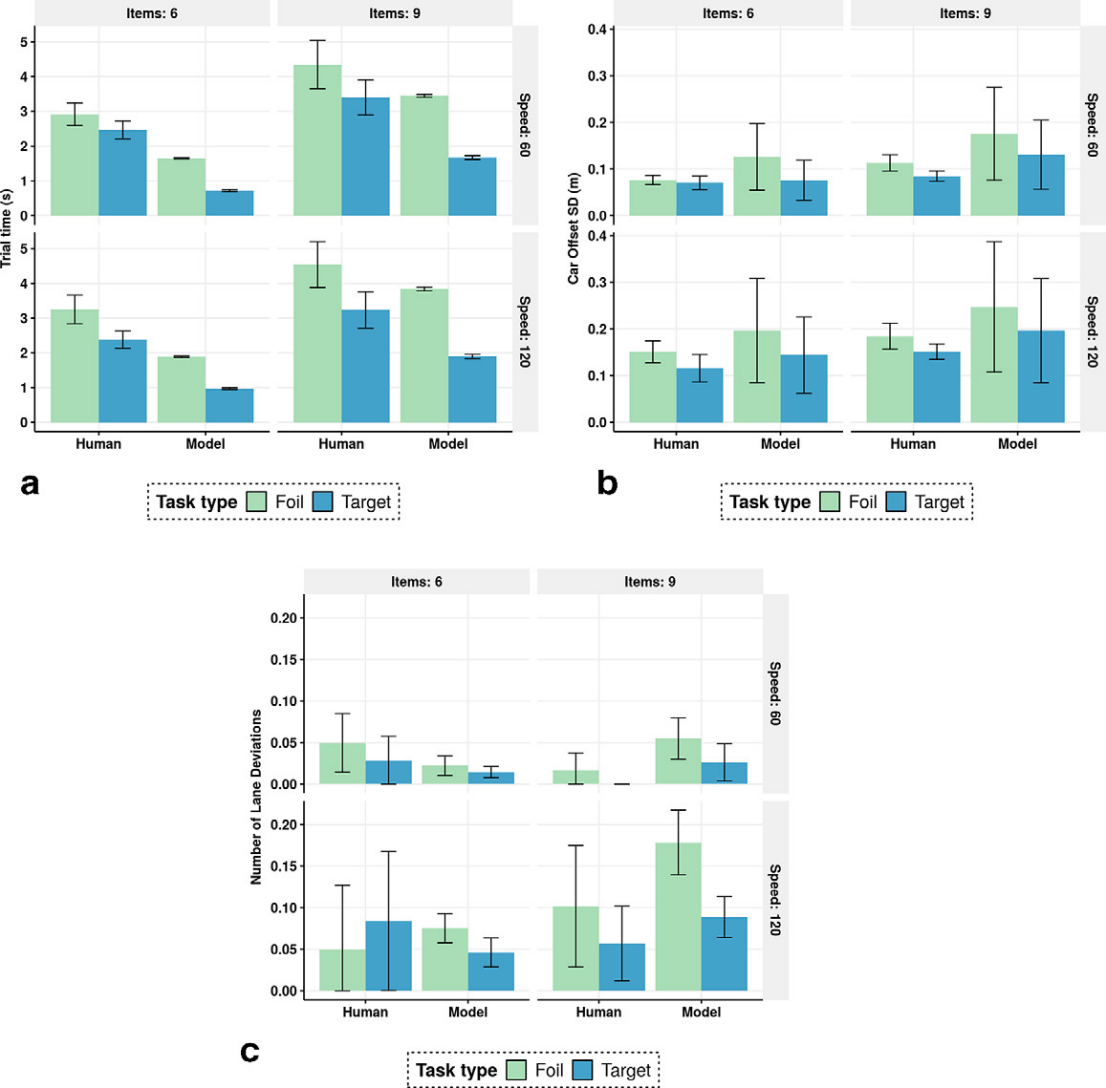
Aggregate values of trial time, car lateral offset, and the number of lane deviations for human data and model prediction. Error bars are standard error with *N* = 12.

**Figure 4:**
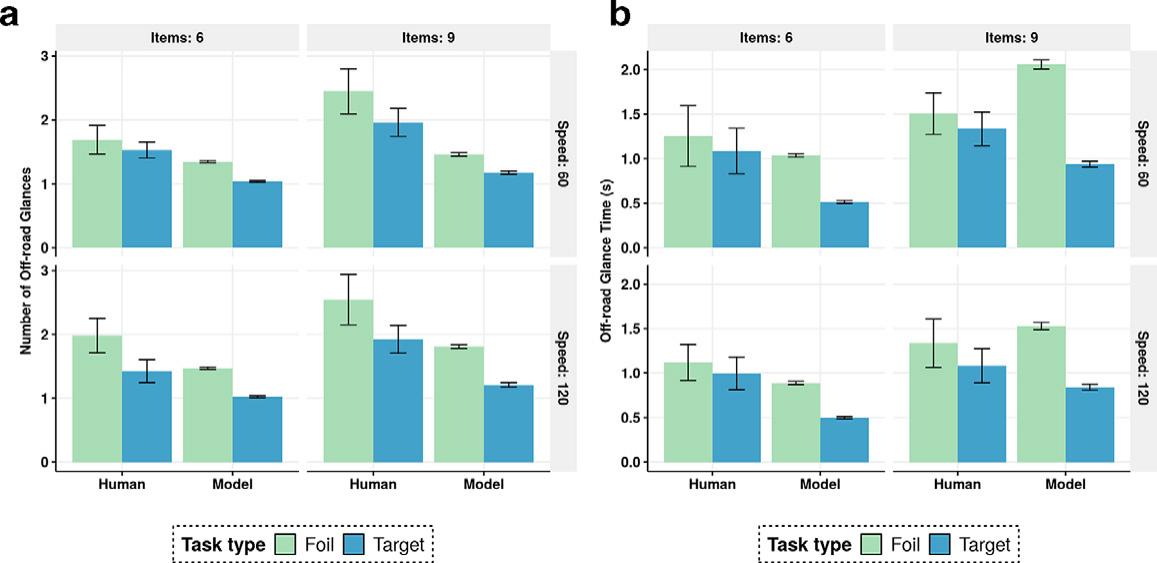
Aggregate values of number and time of off-road glances for human data and model prediction. Error bars are standard error with *N* = 12.

Analyzing the human data, we found statistically significant main effects of the conditions on most of the behavior metrics (see Appendix B, Tables 2–6). In a clear exception, lane deviation was not very dependent on the experimental manipulations; it was affected only by speed (Appendix B, Table 3). This is because the number of lane deviations was typically small, and there was great variation in this metric across participants. The model’s reward function corresponded to the instructions to the participant in that the model tried to hold the car on the road without lane deviations, while conducting visual search tasks. Figures 3 and 4 depict the overall observed (human) and predicted (simulated) values for the key metrics. The effects of the task conditions on the glance metrics are consistent with previous findings (Kujala & Salvucci, 2015; Wierwille, 1993). Also, these figures present new evidence: the number of in-car search items affects all the behavior metrics considered, except lane deviation.

**Table 3:** Multilevel Regression Model for *SD* of Lane Offset

Fixed Effect	Estimate	*df*	*T*
Intercept	0.08	26	9.8***
Speed	0.06	74	10.3***
Task type	−0.03	74	−4.1***
Items	0.03	74	4.8***

*Note*. ***p < .001.

**Table 4:** Multilevel Regression Model Lane Deviation

Fixed Effect	Estimate	*df*	* T*
Intercept	0.03	23	1.4
Speed	0.05	74	3.1**
Task type	−0.01	74	−0.8
Items	−0.01	74	−0.6

*Note*. **p < .01.

**Table 5:** Multilevel Regression Model for Number of In-Car Glances

Fixed Effect	Estimate	*df*	* T*
Intercept	1.8	13	13.6***
Speed	0.1	74	1.2
Task type	−0.5	74	−6.4***
Items	0.5	74	7.6***

*Note*. ***p < .001.

**Table 6:** Multilevel Regression Model for Duration of In-Car Glances

Fixed Effect	Estimate	*df*	*T*
Intercept	1.0	12	8.5***
Speed	−0.0	29	−0.4
Task type	−0.2	29	−2.3*
Items	0.2	29	2.7*

*Note*. *p < .01, ***p < .001.

Our model mirrors the experimental findings. Its average prediction error values, along with regression *R*^2^s, are presented in Table 1. The model’s simulations match the human data fairly closely and replicate almost all of the effects of the experimental manipulations on the performance metrics (apart from lane deviations, as discussed above). The model is faster than humans, as evidenced by shorter trial times (Figure 2a and fewer off-road glances, Figure 3a). This means that the model does not identify all bounds under which the human participants are operating. Also noteworthy are some behaviors that might vary between participants, such as deliberation before confirming the target’s presence or absence, which we did not model.

**Table 1:** Model Fit Indices: Prediction Error in Absolute Terms, Relative to Values From Observed (Human) Data, Standardized to Observed *SD* and Expressed as Linear Model *R*^2^

Metric	Error	Relative Error	Error *SD*	*R* ^2^
Trial time (s)	1.31	0.43	1.63	.95
Offset SD (m)	0.04	0.35	1.15	.88
Lane deviations	0.04	−	1.10	.49
Number of in-car glances	0.60	0.31	1.37	.93
In-car glance duration (s)	0.33	0.29	0.81	.80

**Table 2:** Multilevel Regression Model for Trial Time

Fixed Effect	Estimate	*df*	*T*
Intercept	3.2	13	13.3***
Speed	0.1	74	0.7
Task type	−0.9	74	−8.8***
Items	1.1	74	11.2***

*Note*. ***p < .001.

For the 60 distinct pairwise comparisons for the performance metrics between conditions, the model predicts the direction of the effect incorrectly in only 8 cases, 5 of them involving lane deviations. For example, for a foil task in the high-speed condition, it correctly predicts a positive correlation between a larger search area (nine items vs. six) and car offset (Figure 2b). The high *R*^2^s for all metrics considered indicate also that the relative magnitudes of these changes are correctly predicted (Table 1). As expected, the model generates less variation than was observed in the human data, largely because of fixed-value parameters (see the error bars in Figures 3 and 4). However, because of how the driving task is specified (in terms of discrete actions and position beliefs; see Appendix A, Algorithm 1), the driving task generates greater variance compared to human data. One could tune the free parameters (e.g., of the eye-movement model and driving model) to adjust the model’s search times and driving performance. However, these parameters are always at least somewhat task-dependent, and parameters adjusted to specific tasks would not be interesting beyond those tasks.

## Discussion and Conclusions

In this paper, we have presented a model to improve understanding of a long-standing human factors question: how does a multitasker allocate/shift visual attention between tasks? Our grounding assumption is that task behavior—single or multitasking—can be analyzed as an adaptation to utility, capacity, and ecology. Our model’s goals, such as safe driving, are expressed in terms of a reward function, and the model attempts to maximize its utility by choosing actions that it has learned are rewarding in the long term. This choice is, however, bounded by the capacity of the model. For instance, we add noise to the actions it uses to control the car and observations it makes about the task environment, and stipulate that eye movements between and within tasks take time. Finally, the model is constrained by ecology, that is, its interaction with the task environment, dictated by the simulation of the car movements and the design parameters of the in-car search device.

Within these bounds, we implemented a computational rational agent, which adapts its behavior policy to the task and cognitive constraints to maximize the joint task utility of a driving and an in-car search task. Our simulation-based model is unlike previous models (e.g., Brumby et al., 2007; Kujala & Salvucci, 2015) in applying no prior assumptions about the task-switching policy. Instead, it predicts multitasking strategies as adaptations to task constraints, such as driving speed and in-car interface design, and to learning the tasks’ rewards.

Although we view multitasking behavior as boundedly optimal adaptation, the result is not a model that takes no risks. This is because the model and humans misestimate the environment’s transitions and payoffs, given their limited experience and observability of the situation. More generally, the optimality of the model does not mean that it makes unrealistic assumptions about the human behavior it attempts to simulate. The key is in the definition of the bounds of the agent such that the optimality assumption holds within these bounds. The discrepancies between the model predictions and observed human behavior are due to the underspecification of these bounds. For instance, the model could be augmented to simulate the impact of individual differences, such as expertise, on performance, which would permit matching of predictions to individuals.

While some other models predict the emergence of visual sampling frequencies from informational expectancy and value (Horrey et al., 2006; Wickens et al., 2003), these must be input as constants determined by experts. In our model, the informational expectancy is tied to the inherent uncertainty modeled by POMDPs, and it results from the partial observability of a dynamic simulation environment. The SEEV-AIE model similarly displays successfully derived expectancies; however, these are based on observed event rates in a task model simulation within a cognitive architecture (Wortelen et al., 2013). Importantly, our model simulates driving in varying stages of observability, even full occlusion (Kircher et al., 2018), modeling the variance of task uncertainty that results from partial observability. The model’s multitasking behavior can be characterized as managing driving-related uncertainty, keeping it at acceptable levels when given negative sanctions for poor driving and gains associated with using an in-car interface. More specifically, the model’s behavior can be seen as emerging as a rational adaptation to the bounds of the task environment and those of its own architecture. The model is bounded by its ability to observe only one subtask at a time, by how quickly it can move its eyes and encode targets, and by the noise and speed of its driving actions. Given the rewards specified, such as those related to safe driving and subtask goals, the model adapts its behavior to maximize the long-term joint cumulative task reward within the imposed bounds.

The model was validated against human data without any need to adjust parameters for better fit. All parameters’ values were adopted from established literature or based on the tasks’ specifics. This proves that the model’s fit and the simulated emergence of multitasking strategies were due not to parameter tuning but to the rigorous specification (Roberts & Pashler, 2000). The instructions to the participants were matched to the reward function of the model: lane deviations were discouraged and fast visual search encouraged. Despite no parameter tuning, the model replicates the observed impact of task constraints on multitasking strategies (Table 1). For example, when the number of visual elements in the in-car interface increases, the model attempts to encode more elements within a single glance (Figure 3b). One can explain this observation by appealing to the principle of rational adaptation to task constraints: task switching is costly, so the model (as humans do) maximizes the utility of a glance by devoting more time to searching. Even if this noticeably increases the car’s lateral instability (Figure 2b and c), the joint task utility is greater than in a case wherein the agent opts for several shorter glances instead. Interestingly, in addition, we detected a slight negative interaction effect with speed: when the speed doubles, the number of items has less impact on off-road glance time (Figure 3b). This too is understandable: since the driving task is more demanding, there are fewer resources to devote to adapting to the constraints of the in-car task.

Importantly, our model also predicts unsafe driving behavior resulting from risk-taking, as we observed an increase in lateral instability when the in-car search task showed greater complexity in terms of more search items or faster car speed (Figure 2b and c). Our model can therefore be used to assess the impact of in-car interface design on driving performance. Its general formulation places no constraints on the design or the task, as long as an RL algorithm can approximate an optimal policy connected with it. For instance, the subtask could specify a computer game that the agent tries to play while driving. Further, adding more modalities and their supervisory allocation, such as competing manual actions (e.g., steering the car vs. playing the game) is also supported within our model architecture. Hence, we foresee numerous important applications for our model. In addition to testing the impact of particular in-car tasks and interfaces on safety, we can model the impact of the driving environment. For example, one can model various types of visibility conditions (e.g., night driving or bad weather) by adjusting the observation function, and behavior predictions should unfold as the model adapts to the changes. This also permits the investigation of individual differences (Lee & Lee, 2019), such as driver-specific observation capacity and driving ability.

## Key Points

- Multitasking can be modeled as strategic adaptation to a reward function, for given task-related and cognitive constraints.
- Multitasking strategy manages the uncertainty related to subtasks, attempting to share attentional resources between them to maximize their joint utility.
- A POMDP represents a powerful formalism for modeling and simulating adaptive multitasking behavior.
- A dual-task model of driving and in-car visual search demonstrates that human-like multitasking strategies emerge as rational behavior adapting to tasks and cognitive constraints.

## Appendix A: Algorithms

The driving model first initializes the discrete belief distribution over possible road positions (line 2 of the listing). We discretize 1 m of lateral position into 8 equally wide portions and fix the maximum lateral position to 2 m, which is clearly outside the accepted threshold of 0.875 m. In the beginning, the driver’s belief distribution is uniform over all possible discrete lane positions. Similarly, the action space is defined as a linearly spaced vector, allowing the driving agent to set the steering wheel at maximum to 0.08 radians (4.6°) to left or right (3). We further add an action for 0.0 radian turn, allowing the model to drive straightforward. The first action is set to this action, and the first augmented belief state is set to the most probable lane position of 0.0 and the maximum belief entropy (4 and 5). The transition model is trained by iterating across all lane positions and actions, and using the driving simulator (23) to generate the next position. In the main driving loop, the agent first updates its belief distribution *b_pos_*(*p*) from the transition model using Bayes’ rule (12). Then, if the driver is granted attention for this iteration by the task-interleaving model, the agent observes the true lane position with *P_obs_* and otherwise a random position. We set *P_obs_*=0.9 to reflect high visibility. Again, Bayes’ rule is used to update the belief distribution *b_pos_*(*p*). This distribution is summarized into an augmented belief as the most probable belief and the entropy of the belief distribution (18). Both values are rounded into one decimal point to enforce a limited amount of possible augmented belief states. Softmax action selection policy (τ = 1.0 for learning and τ = 0.1 after model convergence) is used to balance exploration and exploitation (20), and SARSA learning (α = 0.1, γ = 0.9) is used to update the *Q*-values (21). Action-dependent noise *σ_d_* = 0.5 is added to action a′ to get the true steering wheel angle ω. This parameter value was hand-tuned: with value 0.9, the model was able to drive very long with eyes closed, and with the value 0.1, it was not able to keep lane position even with eyes on the road; the value 0.5 was used to balance this. Based on the speed (60 or 120 km/hr), the current lane position, the duration of one driving iteration (0.150 s), and the steering wheel angle, the simulator calculates a new position (23). If the norm of this position is equal to or larger than the lane violation threshold (0.875 m), the model gets a reward of –1, and otherwise 0. The driving model was trained for 700,000 simulated seconds of driving.

The search model is initialized by first creating the observation matrix, one cell for each visual element in the device (2). Each cell is initialized to –1, meaning that it is unobserved. The model is given a random fixation position on the device (but note that this cell is not observed yet). If the randomly selected task type has the target present among the distractor visual elements, one of the elements is randomly assigned as the target. In the main loop, the model observes the visual element under the current fixation, changing the corresponding cell in the observation matrix *obs* from −1 to 0 (11). The belief of the model is the combination of the updated observation matrix and the current fixation location. New action, that is, fixation location, is selected using softmax policy (*T* = 1.0 for learning and *T* = 0.1 after model convergence), and SARSA learning (*α* = 0.1,*γ* = 0.9 ) is used to update the *Q*-values. Reward is initialized to the negative of the EMMA-based movement time. All EMMA parameters are taken from the original publication: *K* = 0.006, *k* = 0.4, *f* = 0.1, *t_prep_*=0.135 , *t_exec_*=0.07 , and *t_sacc_*=0.002 (Salvucci, 2001). In addition, if the target is present in the interface and has just been uncovered, the reward is increased by *R_found_*=20, minus the 0.15 (seconds) for motor response time. This reward value is set high enough to have one visual search task to generally have a net positive reward. Because the trial terminates after the target is found, simple TD(0) learning (19) is used to update the *Q*-values (same parameter values as in SARSA). Alternatively, if the target is not present in the device during this trial, but the model has uncovered all objects, it gets the reward and a new task starts. The search model was trained for 3,200,000 simulated seconds.

The supervisory multitasking or task-interleaving model is initialized with a reference to the driving model and the visual search model, which have been initialized and trained to convergence. In the main loop, the belief state of the model is the combination of the previously selected action *a* and the maximum *Q*-values available for the subtask models, given their current belief (7–10). The best *Q*-values are rounded to one decimal point to make the belief state space finite. Softmax action selection policy (τ = 1.0 for learning and τ = 0.1 after model convergence) is used to balance exploration and exploitation (20), and SARSA learning (α = 0.1, γ = 0.9) is used to update the *Q*-values (21). When the subtasks have been trained, they have τ = 1.0 and no longer update *Q*-values. Both subtask rewards are initialized to zero. If the new action is different from the previous action, there is a task switch. In this case, the driving model is iterated for 0.150 s without attention to account for the saccadic movement time between the tasks (Baloh et al., 1975). If the action is to drive, the driving model is iterated for 0.150 s with attention (Salvucci, 2006). If the action is to search, the search task is iterated once, and the driving model is iterated without attention for the duration of the search model iteration. The cumulative reward from the driving model and the reward of the search model are summed together for rewarding the task-interleaving model. The multitasking model was trained for 200,000 simulated seconds. The model was then run 30 times (independent instances) for the duration of 1,000 s to generate multiple simulations of the model performance within a time frame similar to that of the human participants.

## Appendix B: Hypothesis Tests

All hypothesis test are made using multilevel linear regression models with task conditions as fixed effects and participants as random effects. Models are calculated using R’s lme4 package with lmerTest package for approximating degrees of freedom. The variable *Speed* indicates change from 80 to 120 km/hr. *Task type* indicates change from a “foil” task to a “target-present” task. *Items* refers to a change from six to nine items in the in-car interface.
